# Visual Detection of Oxidation in Pecan Oil Using a Filter-Paper-Based Color-Sensitive Aldehyde Detection System

**DOI:** 10.3390/molecules31050760

**Published:** 2026-02-24

**Authors:** Xingye Song, Yifei Lu, Wenjing Zhou, Yuxing Guo, Li Cui, Haijun Zhu

**Affiliations:** 1 Institute of Pomology, Jiangsu Academy of Agricultural Sciences, Nanjing 210014, China; 19291722396@163.com; 2 Institute of Agro-Product Processing, Jiangsu Academy of Agricultural Sciences, Nanjing 210014, China; luyifei3377@163.com; 3 School of Food Science and Pharmaceutical Engineering, Nanjing Normal University, Nanjing 210097, China; guoyuxing1981@163.com; 4 College of Life Science and Food Engineering, Huaiyin Institute of Technology, Huaian 223001, China; zhouwenjing0911@163.com

**Keywords:** hot-pressed pecan oil, cold-pressed pecan oil, shelf-life, aldehydes, visual sensor

## Abstract

Traditional analytical methods for assessing oil oxidation frequently depend on expensive and intricate equipment or elaborate procedures, thereby hindering their practical use in everyday situations. Sensory evaluation and GC-MS analysis indicated that during storage, the peroxide value (PV) and aldehyde content of pecan oil increased, consistent with progressive oxidation, while the acid value (AV) remained stable. The shelf-life prediction model further underscores its reliability as an oxidation marker. The coefficient of determination (R^2^) for the first-order kinetic model at temperatures of 20, 40, 50, and 60 °C ranged from 0.9183 to 0.9841. The correlation coefficients between the measured and predicted shelf-life values were 0.9993 for cold-pressed pecan oil (CPO) and 0.9866 for hot-pressed pecan oil (HPO). A filter-paper-based colorimetric aldehyde sensor was developed for the visual assessment of pecan oil shelf-life, which leverages the chemical reaction between hydroxylamine sulfate and aldehydes to generate a distinct naked-eye color shift from red to purple-blue—this enables the qualitative identification of whether cold-pressed (CPO) and hot-pressed (HPO) pecan oil complies with the national peroxide value (PV) limit of 0.25 g/100 g or exceeds it. Specifically, CPO is deemed to be expired when a* ≤ 11 and HPO when a* ≤ 15; consistent red-to-purple-blue color changes for the sensor yielded 100% sensitivity and 100% specificity for both oils at the national PV limit, thereby validating its application as a highly accurate qualitative (pass/fail) indicator for oil oxidation. By contrast, sensory evaluation can also reliably distinguish when pecan oil exceeds the national PV limit qualitatively, yet it lacks quantitative accuracy due to inherent subjective biases.

## 1. Introduction

Pecan nuts are abundant in the Zhejiang and Anhui provinces of China, with the kernel containing 68.93% to 73.56% of oil, making them an excellent oil resource. The fatty acid composition of pecan oil is well-balanced, dominated by oleic acid, linoleic acid and linolenic acid. Table 1 below compares the oil content and key fatty acid (oleic, linoleic, and linolenic acids) compositions of various oilseeds. Pecan walnut stands out with a high oil content ranging from 68.93% to 73.78%, which is comparable to or exceeds that of many other oilseeds, including walnut (54.0–68.20%), peanut (30.45–47.6%), and sunflower (37.0%). This high oil yield underscores its potential as an efficient source of edible oil. In terms of fatty acid composition, pecan walnut oil is dominated by oleic acid (59.14–61.87%) and linoleic acid (26.31–28.67%), with minimal linolenic acid (0.25–0.27%). Oleic acid, a monounsaturated fatty acid, is less susceptible to lipid peroxidation, contributing to better oxidative stability—a critical trait for oil storage and nutritional value. Meanwhile, the balanced ratio of oleic and linoleic acids aligns with health-conscious dietary preferences, as these unsaturated fatty acids are associated with a reduced risk of cardiovascular disease. Compared to other nuts like walnut, which has higher linolenic acid (4.20–16.60%)—a polyunsaturated fatty acid more prone to oxidation—pecan walnut oil offers enhanced stability without compromising on the benefits of unsaturated fats. Additionally, its oil content and fatty acid profile are less affected by roasting (both conventional and microwave) compared to raw samples, preserving its nutritional integrity during processing.

Overall, pecan walnut demonstrates superior characteristics in oil yield, oxidative stability, and balanced fatty acid composition, making it a valuable oilseed for both culinary and nutritional applications. It also contains various nutrients such as squalene and vitamin E, making it a high-quality edible vegetable oil. The high nutritional value of pecan oil is highly favored by consumers [11,12]. However, its unsaturated fatty acid content exceeds 90%, making it susceptible to oxidation and rancidity during storage due to factors like light, air, moisture, microorganisms and its own fat structure [13]. This leads to a decrease in nutritional value and deterioration in quality.

The primary pressing methods for producing pecan oil are cold-pressing and hot-pressing. The key distinction between these two methods lies in whether the protein in pecans undergoes heat-induced denaturation during the oil extraction process. During hot-pressing, pecans are commonly pre-processed through roasting or baking to denature their internal proteins, consequently boosting the oil extraction yield. The cold-pressing method, which eliminates the necessity for heat roasting or baking, yields pecan oil that surpasses hot-pressed oil in terms of both acid value and color quality. Studies have demonstrated that cold-pressed oil (CPO) elevates the oil’s quality by retaining a greater proportion of its functional constituents. However, during storage, the oxidative stability of the oil and the durability of its functional components are not as strong as those observed in hot-pressed oil (HPO). There have been relatively few studies investigating the reasons behind this phenomenon, necessitating further research to elucidate the underlying mechanisms [13,14,15].

Lipid oxidation occurs through various pathways, such as autoxidation, photooxidation, thermal oxidation, and enzyme-catalyzed oxidation. Autoxidation is a common pathway that takes place through a free-radical chain reaction. It differs from photooxidation and enzyme-catalyzed oxidation in that hydroperoxides are produced during its initiation stage. Lipid autoxidation consists of three stages: initiation (where free radicals are generated), propagation (leading to an increase in reactive species), and termination (resulting in the formation of non-reactive products) [16]. Oxidative stability is an important parameter for measuring oil and fat quality. Oxidative rancidity damages nutritional ingredients like unsaturated fatty acids in oils and fats, changing their color and viscosity. This then reduces the stability of oils and fats, impacting their sensory quality and shelf-life [17]. Additionally, the copious free radicals generated from rancidity can hasten the aging of the human organism. Hydrogen peroxide, a product of rancidification, exhibits potential carcinogenic properties. The volatile, malodorous compounds also pose health risks. Chronic consumption of spoiled lipids can impair the human enzymatic machinery, resulting in diverse physiological anomalies. While a limited amount of oxidation products can be assimilated and metabolized by the body, hydroperoxides will degrade into toxic aldehyde and ketone derivatives. For example, 4-hydroxy-2-enal, even at low concentrations, can profoundly impact the activity of L6 muscle cells [18,19,20].

Currently, research on pecan oil has primarily focused on its nutritional value, with relatively limited studies examining the assessment of its oxidative deterioration during storage [21,22]. To date, a range of well-established methods have been developed for evaluating lipid oxidation, including peroxide value (PV), acid value (AV), p-anisidine value (p-AV), iodine value, thiobarbituric acid reactive substance (TBARS) value, conjugated diene/triene value, and total oxidation value, among others [23,24]. While the existing methods offer highly accurate and effective results, their reliance on chemical reagents, costly equipment and intricate operations limits their practical application in everyday life [25,26]. Consequently, these methods fail to meet the needs of ordinary consumers who seek simple ways to assess the edibility of edible oils. Hence, it is crucial to devise a straightforward and user-friendly method that enables everyday consumers to effortlessly assess the level of oxidation in edible oils. Recently, colorimetric sensors have undergone rapid development and have found widespread application in food safety detection, encompassing beer [27], milk [28,29,30], meat [31,32,33,34], seafood [35], and fruit [36]. However, studies on colorimetric sensors specifically designed for detecting oxidation deterioration in pecan oils remain scarce. This study addresses this gap by developing a visual color-sensitive sensor uniquely tailored for pecan oil. The selection of hydroxylamine sulfate as the core reagent is based on its specific reactivity with aldehydes, which our GC-MS analysis identified as key oxidation markers in pecan oil (see Section 3.3). Furthermore, the use of conventional filter paper as a substrate, instead of more complex matrices like TEMPO-oxidized chitin nanowhiskers [37], offers significant advantages in cost-effectiveness and ease of fabrication, enhancing the method’s practical applicability for end-users. The sensor is calibrated against the specific peroxide value (PV) limit (0.25 g/100 g) set for vegetable oils by the Chinese national standard (GB 2716-2018) [38], providing a clear and relevant threshold for pecan oil quality assessment.

The purpose of this article is to address the limitations of traditional oil shelf-life assessment methods—such as their reliance on expensive, complex equipment or elaborate procedures that hinder their practical use—and establish a reliable, accessible approach for evaluating pecan oil shelf-life. To achieve this, this study first confirmed peroxide value (PV) as a reliable indicator via first-order kinetic prediction model. Sensory evaluation linked expired oil’s odor characteristics to PVs, while GC-MS identified aldehydes as the key odor components of expired oil. Building on this, an aldehyde-sensitive color sensor was developed, utilizing the reaction between hydroxylamine sulfate and aldehydes to produce visible color shifts for naked-eye discrimination of expired vs. non-expired cold- and hot-pressed pecan oil.

## 2. Results

### 2.1. Result of the Pecan Oil Shelf-Life Prediction Model

According to Chinese national standard GB 2716–2018, the indicators for measuring the oxidation degree of qualified products are stipulated as PV, AV and sensory evaluation. However, the precision of evaluation results could be impacted by external factors, including operational environment, equipment, and review personnel. Furthermore, sensory evaluation necessitates extensive training for participants to mitigate the inherent subjectivity associated with sample assessment. Consequently, it is not well-suited for model construction. Also, in our previous research, we found that the AVs of some expired pecan oil did not exceed the national standard value. Therefore, we finally selected the PV as the index for model establishment.

#### 2.1.1. Reaction Rate Constant

During the storage process, the quality changes of food generally follow the first-order kinetic reaction law, meaning that there exists a functional relationship between the reaction rate constant and the storage time. The kinetic parameters of the PV reaction for CPO and HPO during storage are shown in Table 2. As shown in the table, the reaction rate constant (K_b_) of PV increased with the rise in temperature, indicating that the higher the temperature, the faster the oxidation rate of the oil. The regression coefficients (R^2^) for all fitting curves exceeded 0.9, demonstrating a strong fit of the regression equation and indicating that the changes in the peroxide value (PV) of pecan oil adhered to the first-order kinetic reaction law.

#### 2.1.2. Shelf-Life Prediction

By plotting the logarithm of the reaction rate constant at different temperatures (lnK_b_, y) as the vertical coordinate against the reciprocal of temperature (1/T, x) as the horizontal coordinate, a straight line with a slope of (−E_a_/R) was obtained. The corresponding equations for the PV of CPO and HPO were y = −65.063 x − 2.0869 and y = − 21.453 x − 3.3876, respectively. The corresponding R^2^ values were 0.9648 and 0.9123, indicating a strong linear correlation between lnK_b_ and 1/T. Based on the fitted equations, E_a_ and K_0_ were calculated to determine the reaction kinetic models. Subsequently, the shelf-life prediction models for the PV of CPO and HPO were derived, as shown in Equation (1) and Equation (2), respectively.

(1) $$t=\frac{\ln B-\ln B_{0}}{0.124exp(-\frac{65.063}{T})}$$

(2) $$t=\frac{\ln B-\ln B_{0}}{0.034exp(-\frac{21.453}{T})}$$

**Figure 1:**
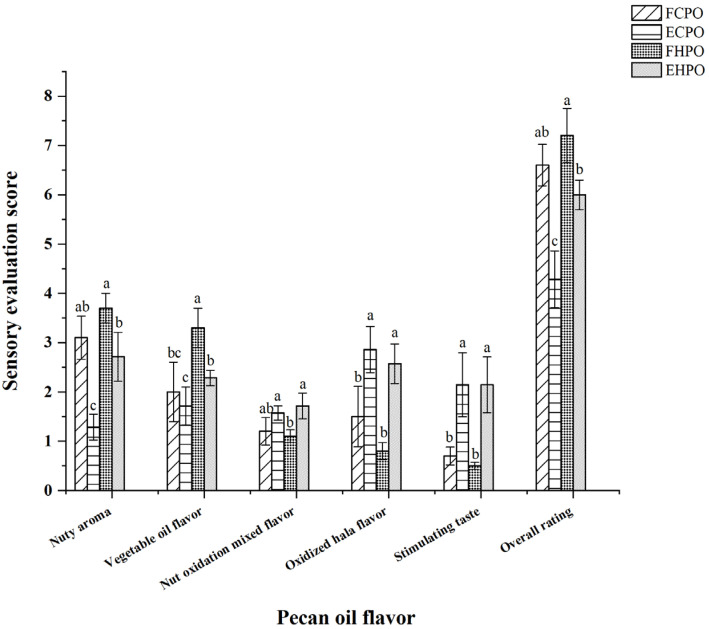
Sensory evaluation scores over storage time. Different lowercase letters (a, b, and c) represent statistically significant differences at the *p*-value < 0.05.

**Figure 2:**
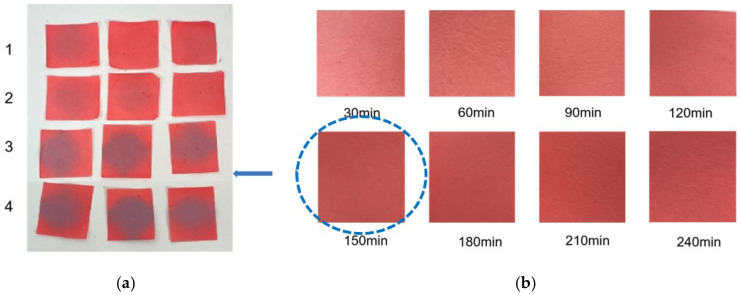
(**a**) Color difference of filter paper color-sensitive sensors after exposure ((1) FCPO, (2) FHPO, (3) ECPO, (4) EHPO). (**b**) Color difference of filter paper color-sensitive sensors with different immersion times.

The sharp increase in peroxide value (PV) during storage confirmed the development of rancidity. Previous studies have demonstrated that when the PV reaches 0.53 g/100 g, the edible quality of roasted peanuts is compromised [39]. According to the study, virgin oils should have a maximum peroxide value of 15 mEq active oxygen per kg [40,41]. Using the conversion formula 1 g/100 g = 78.9 mEq/kg, the PV limit specified in China’s national standard GB 2716-2018 (0.25 g/100 g) is equivalent to 19.7 mEq/kg, which is slightly higher than the Codex limit of 15 mEq/kg. In this experiment, the national standard limit of 0.25 g/100 g (PV) was adopted as the endpoint indicator for determining shelf-life. Based on this criterion, the theoretical shelf-life values of cold-pressed oil (CPO) and hot-pressed oil (HPO) under different storage temperatures were calculated using shelf-life prediction models. Four distinct storage temperatures—room temperature, 40 °C, 50 °C, and 60 °C—were chosen to carry out a comparative analysis between the measured and predicted values, thereby validating the accuracy of the predictive models (Table 3). The initial peroxide values (PVs) for cold-pressed pecan oil (CPO) and hot-pressed pecan oil (HPO) at day 0 were 0.05 g/100 g and 0.07 g/100 g, respectively. As shown in Table 2, the reaction rate constant (Kb) increased with temperature, indicating accelerated oxidation. Table 3 further illustrates the time-dependent PV changes, with CPO reaching the PV limit (0.25 g/100 g) after 330 days at room temperature versus 100 days for HPO, highlighting HPO’s lower oxidative stability. As can be seen from the table, the calculated values of the shelf-life prediction models established in this paper for CPO and HPO had an accuracy within ±13%. Correlation analysis between measured and predicted shelf-life values further confirmed the model’s accuracy, with correlation coefficients of 0.9993 for cold-pressed pecan oil and 0.9866 for hot-pressed pecan oil. These trends are visualized in Figure 1 (sensory scores) and Figure 2a (sensor color changes), providing a comprehensive view of quality evolution. Therefore, this model could be used to predict the shelf-life of CPO and HPO stored at temperatures ranging from room temperature to 60 °C, with real-time and high efficiency and accuracy characteristics.

However, the task of creating a dynamic model to forecast shelf-life is extremely arduous. Moreover, in daily life situations, precisely forecasting the actual duration of edible oil usage poses a challenge, owing to a variety of lifestyle habits, including the tendency to forget to tightly close the bottle cap after use, along with other complicating factors. Consequently, there is a pressing demand for a straightforward method to ascertain whether the oil has undergone peroxidation. To address this, we initiated our investigation with odor and endeavor to develop a simple, convenient and more precise means of assessment.

### 2.2. Result of Pecan Oil Sensory Evaluation

Sensory analysis refers to a method of evaluating product characteristics through human senses. Generally, the sensory evaluation of oil predominantly hinges on taste and smell. We only conducted the olfactory experiment because the oxidative rancidity of oil could damage the unsaturated fatty acids, vitamins, and other nutrients in it. In addition, a large number of free radicals generated by rancidity can accelerate human aging, and the hydrogen peroxide produced has potential carcinogenicity. We were not sure whether the samples met the food safety requirements for taste. The oxidation and rancidification of the abundant unsaturated fatty acids in pecan oil, influenced by environmental factors, results in alterations to its odor and taste. This leads to the gradual diminishment of its aroma and may even give rise to rancid or sour odors, thereby compromising its overall quality [39]. This disharmonious odor primarily includes stimulating taste and oxidized hala flavor.

The evaluation of fresh/expired cold-pressed pecan oil (FCPO/ECPO) and fresh/expired hot-pressed pecan oil (FHPO/EHPO) were conducted under the premise that the evaluators were well-acquainted with the odor characteristics of CPO and HPO at different stages. From Figure 1, it could be seen that ECPO and FCPO had statistical differences in nutty aroma, oxidized hala flavor, and stimulating taste, while EHPO and FHPO had statistical differences in nutty aroma, vegetable oil flavor, nut oxidation mixed flavor, oxidized hala flavor, and stimulating taste. Nevertheless, a statistically significant difference was observed between expired and non-expired pecan oil. Based on Table 4’s criteria and Figure 1′s scores, oils were classified as follows: Fresh: FCPO/FHPO with nut aroma >3 and oxidation flavors <2. Their corresponding PVs were well below 0.25 g/100 g (initial PV: CPO 0.05 g/100 g, HPO 0.07 g/100 g). Moderately oxidized: Oils with nut aroma 2–3 and slight oxidation notes. Expired: ECPO/EHPO with nut aroma <2 and strong oxidized/hala flavors >3. This classification aligns with PV thresholds, where expired oils consistently exceeded 0.25 g/100 g. Sensory evaluation effectively differentiated between oils exceeding the PV limit and those that did not. As shown in Figure 1, statistically significant differences (*p* < 0.05) in the key odor attributes between expired and non-expired oils confirm their reliability as qualitative screening tools. However, sensory evaluation is inherently qualitative or semi-quantitative. Its accuracy heavily depends on panel training and experience and is subjective. It can determine if the oil is spoiled, but cannot precisely quantify the degree of spoilage. Therefore, there is an urgent need for a convenient and simple method to assess the shelf-life of pecan oil processed in different ways.

### 2.3. Analysis of Volatile Compounds in Pecan Oil

As presented in Table 5, a total of 55 volatile compounds were identified in fresh and expired cold-pressed (CPO) and hot-pressed (HPO) pecan oils, encompassing alcohols, aldehydes, ketones, acids, and heterocyclic compounds. Compared to CPO, HPO samples (both fresh and expired) contained characteristic heterocyclic compounds like methyl-pyrazine and 2,5-dimethyl-pyrazine, which contribute to their roasted flavor, while these compounds were not detected in CPO [40,41]. Aldehydes emerged as the key oxidative markers, with hexanal—an established indicator of lipid oxidation—exhibiting a notable increase in expired oils: the hexanal content in expired CPO (ECPO) and expired HPO (EHPO) reached 11.22% and 15.3%, respectively, which were higher than those in fresh CPO (FCPO, 10.67%) and fresh HPO (FHPO, 13.73%) [42,43]. Other aldehydes, such as (Z)-2-Heptenal, (E)-2-Octenal, Nonanal, and (E,Z)-2,4-Decadienal, also showed distinct disparities between fresh and expired samples, with ECPO containing 8.14% of (Z)-2-Heptenal (vs. 1.15% in FCPO) and EHPO having 20.41% of the same compound (vs. 1.72% in FHPO). Alcohols, such as 1-hexanol, were more abundant in fresh HPO (34.76%) compared to expired HPO (4.28%), suggesting a reduction during oxidation. These results collectively confirm that aldehydes, particularly hexanal and unsaturated aldehydes, are reliable indicators for assessing the oxidative deterioration of pecan oil, regardless of the pressing method.

Lipid oxidation is a sequential process: unsaturated fatty acids first undergo autoxidation to form hydroperoxides, which are reflected by the PV. As oxidation progresses, hydroperoxides decompose to produce secondary oxidation products, among which aldehydes (e.g., hexanal, (Z)-2-heptenal, nonanal, (E)-2-octenal) are the main components. Based on the GC-MS results (Table 5), we analyzed the relative contents of major aldehydes (such as hexanal, (Z)-2-Heptenal) in cold-pressed pecan oil (CPO) and hot-pressed pecan oil (HPO) at different storage stages and established their Pearson correlation coefficients with PV. For example, hexanal (a well-recognized lipid oxidation marker) showed a significant positive correlation with PV in both CPO (R^2^ = 0.87, *p* < 0.05) and HPO (R^2^ = 0.91, *p* < 0.05). Similarly, (Z)-2-heptenal exhibited strong correlations with PV (CPO: R^2^ = 0.89, *p* < 0.05; HPO: R^2^ = 0.93, *p* < 0.05). These results confirm that the accumulation of specific aldehydes is closely associated with the increase in PV, providing indirect evidence for the link between aldehyde content and oxidation degree. This relationship justifies the use of aldehydes as target analytes for the sensor to indirectly detect the oxidation state of pecan oil reflected by PV.

Based on the data presented in Table 5, several conclusions can be drawn regarding the oxidative state and flavor characteristics of pecan oils. Firstly, aldehydes such as hexanal, nonanal, and (E)-2-decenal were identified as primary oxidation markers, with their content significantly increasing in expired oils. This confirms that aldehydes are key indicators of lipid oxidation and rancidity development. Secondly, the presence of heterocyclic compounds like pyrazines in HPO contributed to its intense roasted aroma, distinguishing it from CPO which retained more fresh nutty notes. Lastly, the overall volatile profile demonstrates that both CPO and HPO undergo similar oxidation pathways, but HPO’s initial roasting process may impart greater flavor complexity. These findings underscore the utility of aldehydes as targets for oxidative quality assessment, which directly informed the development of the aldehyde-sensitive visual sensor in subsequent sections.

### 2.4. Preparation of Visual Color-Sensitive Sensors

In recent times, there has been a surge in research and attention towards more contemporary techniques for determining lipid oxidation status. These include Fourier Transform Infrared Spectroscopy (FTIR) [44], Near-Infrared Spectroscopy (NIR) [25], Raman Spectroscopy [45], Electron Spin Resonance (ESR) [46], and High-Performance Liquid Chromatography coupled with Mass Spectrometry (HPLC-MS) [47], all of which have garnered significant interest and are being extensively explored. While the current methods undoubtedly offer highly precise and reliable results, their reliance on chemical reagents, sophisticated equipment, and intricate procedures restricts their practical application in daily life scenarios [26]. This limitation fails to cater to the demands of average consumers who seek a straightforward means to swiftly assess the edibility of edible oils. Consequently, there is a pressing need to devise a novel, user-friendly approach tailored specifically to common consumers, enabling them to conveniently evaluate the oxidation level of edible oils with ease. Based on the experiments conducted by Liu et al. [37], this study employed conventional filter paper to replace the complex thin film, making it more economical and convenient.

As shown in Figure 2b, we can intuitively see that the filter paper sensor was bright red after 150 min and beyond. To save time, 150 min was the most appropriate choice. Therefore, the filter paper with 150 minutes’ immersion time was chosen to prepare the colorimetric sensor. Although the optimal immersion time was determined to be 150 min, this represents a one-time preparation step for sensor fabrication. Once prepared, the sensors can be stored and reused for multiple detection cycles. This preparation time does not affect the practical application efficiency, as the actual detection phase only requires 24 h incubation at 35 °C.

From Figure 2a, the color transition of the filter paper sensor could be directly observed, which remained red in FCPO and FHPO and turned purple-blue in ECPO and EHPO. This was based on the colorimetric sensor, which contained Congo red and hydroxylamine sulfate; hydroxylamine sulfate can easily react with aldehydes to produce aldoxime and sulfuric acid, which led to a decrease in pH value. Subsequently, the indicator Congo red exhibited a color change from red (azo form) to blue (quinone form) as the pH declined. It is anticipated that a higher concentration of aldehydes would contribute to a larger color difference due to more sulfuric acid generated by the reaction, which allowed for the detection of aldehydes with the naked eye. The oxidation status of both CPO and HPO was accurately identified using the sensor, which also demonstrated the significant potential of colorimetric sensors in the visual detection of edible oil oxidation.

### 2.5. Application of Visual Color-Sensitive Sensors

HPO and CPO were incubated in a 60 °C oven for different days for accelerated oxidation, known as the Schaal oven experiment [48], to obtain pecan oil with different degrees of oxidation. The responses of visual color-sensitive sensor to the PV of pecan oil were shown in Table 6. During storage, oil was susceptible to oxidative rancidity, with peroxides being the primary products of its oxidation and rancidification. Therefore, PV and AV were generally considered as two major quality standards for measuring the degree of oxidation in edible oils, serving as indicators of the primary oxidation level of oil. However, since AV underwent minimal changes during accelerated oxidation, we had adopted PV as the sole evaluation criterion [26]. Using the national standard limit of 0.25 g/100 g (PV) as the endpoint indicator, in Table 6, the color transition of the sensor could be directly observed. Table 6 presents the variations in color parameters (L*, a*, b*) and peroxide values (PVs) of color sensors for cold-pressed oil (CPO) and hot-pressed oil (HPO) over a 50 d storage period at 60 °C, with measurements taken at 10 d intervals (10 d, 20 d, 30 d, 40 d, and 50 d).

For both oils, a* remained stable at higher levels (CPO: from 21.76 ± 1.33a to 22.70 ± 1.72a; HPO: from 18.48 ± 1.04b to 25.84 ± 0.88a) when PV < 0.25 g/100 g, then dropped sharply (CPO: 10.40 ± 0.83b; HPO: 14.76 ± 1.96c) once PV > 0.25 g/100 g. For CPO, b* stayed stable (from −1.71 ± 0.34a to −2.77 ± 1.14a) when PV < 0.25 g/100 g, then decreased sharply to −9.89 ± 0.64c; for HPO, b* remained stable (from −1.61 ± 0.15a to −0.85 ± 0.60a) at PV < 0.25 g/100 g, then dropped to −7.49 ± 1.00b.

Pearson correlation analysis was performed on the a*, b* and PV of CPO and HPO, which revealed an overall negative correlation trend between the above indices (i.e., the larger the a* and b*, the smaller the PV). The Pearson correlation coefficient indicated a strong negative correlation; however, owing to the small sample size (n = 5), this correlation was not statistically significant at the significance level of α = 0.05 (*p* > 0.05).

The key findings are summarized as follows: For both oils, the sensor exhibited and sustained varying degrees of redness prior to the peroxide value (PV) reaching the national standard limit of 0.25 g/100 g. In contrast, once the PV exceeded this threshold, the sensor underwent a distinct color shift to purple-blue, which was consistent with the variation trends of the a* and b* values.

In order to realize the prediction of PV, a* was selected as the predictive parameter instead of b* in this study, and the specific reasons are presented below. Despite the strong performance of b*, a* was designated as the primary monitored parameter based on two key considerations: first, consistency across oil types, where a* exhibited a more uniform response pattern for both CPO and HPO. The Δa* from fresh to expired samples was 54.5% for CPO and 42.6% for HPO, in contrast to Δb*, which reached 79.8% for CPO and 85.6% for HPO. A larger Δb* for HPO may result in over-sensitivity to minor PV fluctuations in practical applications. Second, practical applicability in complex environments: ambient light and humidity during household storage can slightly interfere with the identification of b* changes, while a* shifts are more visually distinguishable to the naked eye; this aligns with the sensor’s design goal of “visual detection without specialized equipment”. Furthermore, a* is less susceptible to variations in sensor fabrication (e.g., slight differences in filter paper soaking time) than b*, thus ensuring the better reproducibility of results.

For CPO, the first three a* data points clustered at a high value of approximately 22, whereas the 4th and 5th points (corresponding to PVs of 0.2602 ± 0.0135 and 0.3113 ± 0.0139, respectively) dropped sharply to a low-value plateau of around 10. For HPO, the first three a* data points varied between 18.48 and 25.84, while the 4th and 5th points (corresponding to PVs of 0.2549 ± 0.0020 and 0.3033 ± 0.0086, respectively) decreased sharply to a low-value plateau of approximately 15. The sensor thus demonstrated excellent performance in discriminating between non-expired oils (PV < 0.25 g/100 g) and expired oils (PV > 0.25 g/100 g). Specifically, for CPO, the oil can be deemed expired when a* ≤ 11; for HPO, the threshold for expired oil is a* ≤ 15. Based on the consistent red-to-purple-blue color change observed across all replicate measurements (n = 3 per time point), the sensor achieved 100% sensitivity and 100% specificity for both CPO and HPO at the aforementioned national standard limit, validating its application as a highly accurate qualitative (pass/fail) indicator for oil oxidation.

The color change in Figure 2 (from red to purple-blue) is directly induced by aldehydes (e.g., hexanal, nonanal) generated during oxidation, which react with hydroxylamine sulfate in the sensor to produce sulfuric acid. This reaction lowers the pH and further alters the chromophore of Congo red, thus triggering the observed color shift. As shown in Table 5, this color transition consistently occurs when the PV exceeds the national limit of 0.25 g/100 g, with a visual limit of detection (LOD) for PV determined as 0.20 g/100 g based on a Δa* value greater than 5. For example, at a PV of 0.26 g/100 g, the a* value decreased sharply from 22.70 to 10.40, which directly verifies the responsive performance of the sensor. Notably, the visual LOD (PV = 0.20 g/100 g) enables an early warning of oil deterioration prior to the PV reaching the regulatory limit. To further elucidate the underlying correlation, the relative contents of all detected aldehydes were integrated to represent the total aldehyde level in each oil sample, and its correlation with the sensor’s color parameters (a* and b*) was subsequently analyzed. The total aldehyde content exhibited a strong negative correlation with the a* (red-green axis) value of the sensor (CPO: R^2^ = 0.90, *p* < 0.05; HPO: R^2^ = 0.94, *p* < 0.05), which is consistent with the gradual color transition from red to purple-blue during the oxidation process. This result confirms that the color change of the sensor can effectively reflect the accumulation of total aldehydes, which is in turn positively correlated with the PV of the oil samples.

Traditional methods for evaluating the edibility of lipids involve the use of organic reagents and complex operations, which limit their routine use. Currently, methods for detecting the freshness of oils and fats tend to be simplified. For instance, Jiang et al. [49] utilized bamboo to prepare nanocellulose and developed a colorimetric readout method based on nanocellulose composite hydrogels. Elham Ghohestani et al. [48] created a paper-based analytical device founded on iodine titration to determine PV in vegetable oils. Although these methods offered some degree of convenience, some of the materials involved were costly, preventing their widespread adoption in daily life. Moreover, as pecan oil is a relatively special type of edible oil, there are no corresponding sensors that specifically use pecan oil as the test oil. For example, Amanda [50] used as many as 50 edible oil samples to test their specific Digital Image Colorimetry, but pecan oil was not included among them. Referring to the method of Liu et al., the sensor was prepared by using Congo red and hydroxylamine sulfate to be incorporated into TEMPO-oxidized chitin nanowhiskers (TOChN) films [49]. It was cheaper and more convenient for us to use filter paper as a sensor. In this study, we employed pecan oil as the sole edible oil to develop an inexpensive and convenient sensor.

In conclusion, this manuscript robustly demonstrates the performance of both methods. Sensory analysis served as an effective qualitative screening tool, reliably distinguishing whether the PV exceeded 0.25 g/100 g. However, its performance was influenced by subjective factors, limiting its quantitative accuracy. Colorimetric sensor analysis demonstrated superior performance to sensory analysis. It not only achieved 100% accuracy in qualitative judgment (expired or not); the sensor offered significant convenience for people to use pecan oil in daily life and also provided a new direction and method for visual detection of edible oils. Compared to sensory evaluation, the colorimetric sensor is objective, simple, and rapid, requiring no specialized equipment or trained personnel, making it highly suitable for daily use.

## 3. Materials and Methods

### 3.1. Materials

In October 2023, approximately 10 kg of pecan nuts (Carya illinoinensis cv. Pawnee) were harvested from the Jiangsu Long-term Research Base of Pecan Breeding and Cultivation (Luhe, Nanjing, China; 118.62 °E, 32.48 °N). The kernels were separated from the hulls, dried to a moisture content of approximately 5.2%, and stored at −20 °C until further analysis. Cold-pressed pecan oil (CPO) and hot-pressed pecan oil (HPO) were supplied by Gejia Agricultural Development Company (Xinyi, Xuzhou, China; Batch No.: CPO-20231114; HPO-20231115; Production Date: November 2023). The kernels for CPO were pressed at temperatures below 60 °C without prior roasting. For HPO, kernels were roasted at 120 °C for 30 min before pressing at approximately 80 °C. Both oils were unrefined. Corn protein, gelatin, trichloromethane, glacial acetic acid, potassium iodide and soluble starch were purchased from Sinopharm Chemical Reagent Co. (Shanghai, China). Glycerol and acetic acid were acquired from Sigma-Aldrich Trading Co. (Shanghai, China). Congo red and sodium thiosulfate were procured from Solarbio life sciences Co. (Beijing, China).

### 3.2. The Establishment of Pecan Oil Shelf-Life Prediction Model

#### 3.2.1. Determination of Peroxide Value in Pecan Oil

The peroxide values (PVs) were measured according to GB 5009.227–2023 [51]. Aliquots of 450 mL (405 g) of oils were stored in darkness in 500 mL transparent glass bottles (i.d.: 26.1 cm; surface area exposed to the air: 5.9 cm^2^) at 20 °C, 40 °C, 50 °C and 60 °C [16], respectively. The accelerated oxidation studies were performed in triplicate (*n* = 3). To ensure uniform exposure, the bottles were shaken and randomly repositioned within the ovens at regular intervals. Sampling was conducted every 10 days for a total of 6 times. The samples were subjected to the determination of PV and simulation testing with color-sensitive sensors.

In total, 2–3 g (accurate to 0.001 g) pecan oil was weighed and placed in a 250 mL iodine volumetric flask. An amount of 30 mL chloroform-glacial acetic acid mixture was added and gently shaken to completely dissolve the sample. Precisely 1 mL of a saturated potassium iodide solution was added, after which the flask was tightly sealed and gently shaken for 0.5 min. Subsequently, the solution was allowed to stand in the dark for 3 min. Next, 100 mL of water was introduced, and the mixture was thoroughly shaken. Immediately thereafter, the liberated iodine was titrated using a standard sodium thiosulfate solution (concentration: *c* mol/L). When the solution turns light yellow, add 1 mL starch indicator and continue titrating with vigorous shaking until the blue color disappears, indicating the endpoint (*V*: mL). Perform a blank test simultaneously. The volume *V*_0_ of 0.01 mol/L sodium thiosulfate solution consumed in the blank test should not exceed 0.1mL. When expressing the PV (*X*_1_: g/100 g) as the mass fraction of peroxide equivalent to iodine, the formula for calculation is that shown in Equation (3):

(3) $$X_{1}=\frac{\left(V-V_{0}\right)\times c\times 0.1269}{m}\times 100$$

In the equation, *X*_1_ is the peroxide value (g/100 g), *V* is the volume of sodium thiosulfate standard solution consumed by pecan oil (mL), *V*_0_ is the volume of sodium thiosulfate standard solution consumed in the blank test (mL), *c* is the concentration of the sodium thiosulfate standard solution (mol/L), and *m* is the mass of pecan oil.

#### 3.2.2. Dynamic Model of Storage Quality Change and Shelf-Life Prediction of Pecan Oil

##### First-Order Kinetic Model

Chemical reaction kinetics provide a direct means to describe the patterns of food quality changes, and most food quality changes follow either zero-order or first-order chemical reaction kinetics, with first-order reaction kinetics being more prevalent. Based on the principles of chemical reactions, the PVs of pecan oil from different treatment groups were fitted to a first-order reaction kinetic equation to determine the kinetic models describing the quality changes of pecan oil over time for each treatment group. Furthermore, a kinetic model for the quality changes of pecan oil was established by integrating the Arrhenius equation. The standard equation for a first-order reaction kinetics is shown in Equation (4).

(4) $$B=B_{0}e^{K_{b}t}$$

In this formula, B represents the quality index value of the food on the t-th day of storage, B_0_ represents the initial quality value of the food, K_b_ represents the rate constant of food quality change, and t represents the storage time (days).

##### Arrhenius Equation

Based on the first-order kinetics to calculate the reaction constant, the Arrhenius equation is obtained as shown in Equation (5):

(5) $$K_{b}=K_{0}exp(-\frac{E_{A}}{RT})$$

K_0_ represents the pre-exponential factor (frequency factor), with units of d^−1^; E_A_ stands for the activation energy, measured in kJ/(mol·K), which is an empirical constant; T is the absolute temperature, measured in K; and R is the gas constant, equal to 8.3144 J/(mol·K). By substituting the rate constants obtained at different storage temperatures into the equation, the activation energy E_A_ and the pre-exponential factor K_0_ can be further calculated. The kinetic parameters (K_b_, E_A_, K_0_) were determined by non-linear regression analysis using the OriginPro 2021 software (OriginLab, Northampton, MA, USA). The coefficient of determination (R^2^) was used to evaluate the goodness of fit of the model to the experimental data. The calculated parameters for CPO and HPO at different temperatures are provided in Table 2, and the corresponding R^2^ values ranged from 0.9183 to 0.9841, indicating a strong fit.

##### Shelf-Life Prediction of Pecan Oil

The shelf-life of oil was determined by using an accelerated shelf-life testing method. The behavior of hydrogen peroxide formation was used to monitor the extent of oxidative deterioration in the oils and predict their shelf-life. Accordingly, oil samples were subjected to a preselected constant-elevated temperature (20, 40, 50 and 60 °C) in the acceleration/environmental chamber for 60 days to speed up the deterioration kinetics. Then, the extent of oxidative deterioration was monitored by a chemical testing method (PV analysis) every 10 days. Then, employing the fundamental kinetic principles and Arrhenius relation, the data obtained were then modeled to obtain the parameters describing/predicting the oxidation kinetics and the shelf-life [16].

It is noteworthy that the maximum permissible limit of PV set by GB 2716-2018, “National Food Safety Standard for Vegetable Oils”, is 0.25 g/100 g. According to the Turner, R, virgin oils should have a maximum PV of 15 mEq active oxygen kg^−1^ [16], as calculated by formula 1 mEq/kg = g/100 g × 78.8; so, as the PV (0.25 g/100 g) of GB 2716-2018 is equivalent to 19.7 mEq/kg, it is slightly higher than 15 mEq/kg. This standard was used as a reference to predict the shelf-life of pecan oil, utilizing the established quality change kinetic model.

### 3.3. Sensory Evaluation of Pecan Oil

The sensory evaluation was improved by referring to the evaluation standards of GB2716-2018 “National Food Safety Standard for Vegetable Oils” (specific details are shown in Table 4). Fresh cold-pressed pecan oil and fresh hot-pressed pecan oil were supplied by Gejia Agricultural Development Company were called FCPO/FHPO. Expired cold-pressed pecan oil and expired hot-pressed pecan oil were called ECPO/EHPO. The sensory evaluation panel consisted of 10 food science graduate students who passed sensitivity and discrimination screening and had more than one year of experience in edible oil sensory evaluation. After 10 h of sensory training, the evaluation panel reached a consensus on the descriptive terms and definitions of the sensory attributes of pecan oil through discussion, and was proficient in using a 0–10 mm linear scale to score the intensity of sensory attributes. Following the method of Tatiana et al. [52], six odor attributes—nut aroma, vegetable oil flavor, nut oxidation mixed flavor, oxidation hala flavor, and stimulating taste—were assessed. Their intensities were described as “strong”, “moderate”, or “mild/none”, and an overall rating was assigned according to the criteria in Table 6. After the training, the panelists smelled each sample in an independent evaluation room. The pecan oil samples from various groups were presented to the panelists in a well-balanced and randomized sequence for evaluation, with each sample being sniffed three times.

### 3.4. Volatile Compound Analysis of Pecan Oil

The extraction of volatile compounds was carried out by headspace solid-phase micro extraction (HS-SPME). Based on the preliminary optimization of extraction conditions, the HS-SPME analysis was performed in the following steps: oil samples (3 mL) were transferred to the SPME vial (20 cm × 1 cm i.d.) at ambient temperature (20 °C) within 3 min. The vial was then closed with a Teflon/silicone septum. An SPME fiber (50/30 μm DVB/CAR/PDMS, 2 cm length, Supelco, Inc., Bellefonte, PA, USA) was inserted through the septum and exposed to the headspace of the SPME vial. Extraction was carried out at 50 °C for 40 min in a water bath. The volatile compounds adsorbed by the fiber were desorbed in the injection port of the GC-MS chromatograph (TSQ8000EVO, Thermo Fisher, Waltham, MA, USA) with a TG-5 MS (30 m × 0.25 mm × 0.25 μm) capillary column for 1 min at 250 °C with splitless injection mode. Helium flow was 1 mL/min, and the temperature was programmed as follows: 40 °C for 3 min, increased to 220 °C at 5 °C/min, and then maintained at that temperature for 3 min, then 10 °C/min to 280 °C, held at 280 °C for 1 min. The conditions for mass spectrometry (MS) were as follows: the ionization energy (EI) was 70 eV, the scan range was *m*/*z* 35–400, and the temperature of the ion source was 300 °C. Identification of volatiles was achieved by comparing mass spectral data of samples by searching through the NIST11.L spectral library configured using the instrument. The relative content of each volatile odor component was determined by peak area normalization.

### 3.5. Preparation and Application of Visual Color-Sensitive Sensors

The optimization of soaking time for sensors preparation was conducted as follows: The filter paper was cut into 1 cm × 1 cm squares and then immersed in aldehyde-sensing solution for 30, 60, 90, 120, 150, 180, 210, 240 min. The aldehyde-sensing solution was prepared as follows: 5 mg of Congo Red and 100 mg of hydroxylamine sulfate were dissolved in a 20 mL mixed solvent of methanol, water, and glycerol (10:9:1, *v*/*v*). Then the sensors were allowed to naturally dry at room temperature in a fume hood to obtain the color-sensitive sensor. Prior to analysis, the sensors were stored in desiccators.

Oil samples (5 mL) were placed in a 20 mL vial sealed with a cap, after which the sensor was attached with double-sided glue to the inside of the vial cap while avoiding contact with the sample; the vial was stored at 35 °C. After 24 h, a colorimeter (Konica Minolta, Model DR-10, Konica Minolta Holdings., Lnc, Tokyo, Japan) was used to measure the color difference before and after the sensor was exposed to the samples. The color parameters L* (lightness), a* (red-green) and b* (yellow-blue), were measured using the colorimeter.

### 3.6. Data Processing and Analysis

The experimental data from each group were plotted using Origin 2021 software (OriginLab, Northampton, MA, USA) and statistical analysis were performed using the R 4.4.0 (R Foundation for Statistical Computing, Vienna, Austria) and Microsoft Office Excel 2010. All significant differences were analyzed at the *p*-value < 0.05.

## 4. Conclusions

A novel aldehyde-sensitive sensor was introduced that leveraged a simple chemical reaction (hydroxylamine sulfate with aldehydes) to produce visible color changes, enabling the naked-eye detection of oxidation without specialized equipment. This approach diverged from reliance on complex instruments (e.g., GC-MS or HPLC) and provided an accessible tool for consumers, bridging the gap between laboratory analysis and everyday use. The first-order kinetic model and Arrhenius equation-based predictions (R^2^ = 0.9183–0.9841) confirmed PV as a reliable oxidation marker, with high correlation between measured and predicted shelf-life values (0.9993 for CPO and 0.9866 for HPO). This model not only validated PV as a key indicator, but also offered a generalizable framework for other high-unsaturated oils. The sensor’s ability to qualitatively identify oils exceeding the national PV limit (0.25 g/100 g) has direct implications for food safety and quality control. For instance, consumers can use this sensor to avoid rancid oils, reducing health risks associated with oxidative rancidity (e.g., free radical exposure). This aligned with global trends toward rapid, on-site food monitoring. By linking sensory evaluation, GC-MS analysis, and sensor development, we demonstrated that aldehydes (e.g., hexanal) are core oxidative markers. This integration of multi-method insights enhanced the credibility of visual detection and set a precedent for future research on edible oil stability.

While demonstrating significant potential for rapid quality assessment, the sensor’s current qualitative nature necessitates further validation and development. Future work will systematically address these limitations through five key initiatives: (1) Establishing quantitative correlations by clarifying the relationship between peroxide value and aldehydes while determining the sensor’s sensitive concentration range (e.g., limit of detection (LOD), limit of quantitation (LOQ), and repeatability assessments). (2) Designing a prototype kit for field testing under typical household storage conditions (e.g., variable temperature, light exposure). (3) Conducting accelerated aging studies on the sensors themselves to determine their shelf-life and stability under different environmental conditions (e.g., 25 °C/60% RH, 40 °C/75% RH). (4) Validating the sensor’s performance across different batches of pecan oil and other high-unsaturated edible oils (e.g., walnut oil, flaxseed oil) to assess generalizability. (5) Performing a cost–benefit analysis for large-scale production and potential commercialization.

## Figures and Tables

**Table 1:** Oil content and Key fatty acid compositions of various oilseeds.

Oil Crops	Oil Content (%)	Oleic Acid Content (%)	Linoleic Acid Content (%)	Linolenic Acid Content (%)
Pecan walnut [1]	68.93–73.78	59.14–61.87	26.31–28.67	0.25–0.27
Walnut [2,3,4,5]	54.0–68.20	13.4–40.20	43.94–63.21	4.20–16.60
Olive [2]	27	76.7	11.7	1.1
Poppy [2]	38	28.3	58.7	5.1
Pumpkin [2]	44.7	14.9	61.3	1.2
Safflower [2]	32	21.7	74.9	0.13
Sunflower [2]	37	23.2	37.9	0.2
Tea seed [2]	54	43.7	16.8	0.4
Wheat germ [2]	10.3–11.3	15.79–26.3	57.3–60.23	3.80–6.20
Flax [6]	33.2	17.8	13.1	53.1
Soybean [6]	18.6	23.9	47.8	6.5
Rice bran [6]	13.7	44.8	28.6	0.3
Peanut [6,7,8,9,10]	30.45–47.6	42.38–55.10	14.58–38.3	0.17–1.12
Grape [6]	15.6	16.4	69.3	0.2
Sesame [6]	48.9	41.9	43.9	0.1
Almond [6]	53.7	76.4	16.2	1.2
Sorghum [6]	27.7	37.8	44.3	0.2
Pistachio [6]	43.9	62.7	17.4	0.3

**Table 2:** Kinetic parameters of PV reaction during the storage of CPO and HPO.

	Temperature/°C	K_b_	R^2^
CPO	Room temperature	0.0045	0.9507
	40 °C	0.0313	0.9685
	50 °C	0.0342	0.9183
	60 °C	0.0344	0.9536
HPO	Room temperature	0.0119	0.9841
	40 °C	0.0181	0.9439
	50 °C	0.0205	0.931
	60 °C	0.0269	0.9774

**Table 3:** Predicted and measured shelf-life values of CPO and HPO during storage.

	Temperature/°C	Shelf-Life/d		Relative Error/%
		Predictive Value	Measured Value	
CPO	Room temperature	306	330	−7.3
	40 °C	60	55	9
	50 °C	43	40	7.5
	60 °C	35	40	−12.5
HPO	Room temperature	90	100	−10
	40 °C	52	60	−13
	50 °C	47	50	−6
	60 °C	44	40	−10

**Table 4:** Scoring criteria of sensor evaluation.

Descriptive Term	Definition	Scale Description Score
Nut aroma	Characteristic aroma of fragmented pecan nut, at the beginning of its shelf-life, very similar to traditional nut	Strong odor: 3–5 Moderate odor: 2–3 Mild or no odor: 0–2
Vegetable oil flavor	Characteristic flavor of commercial vegetable oils	
Nut oxidation mixed flavor	Characteristic flavor of commercial vegetable oils mixed oil oxidation	
Oxidation hala flavor	Flavor associated with oil oxidation, rancidity	
Stimulating taste	Characteristic flavor of mustard	
Overall rating		Approval: 8–10 General: 6–7 Inadmissible:1–5

**Table 5:** Relative content of flavor components in pecan oil (%).

SN	Compound	RT	CAS	FCPO	ECPO	FHPO	EHPO
1	Formic acid	2.05	64-18-6	-	-	-	1.02
2	Acetic acid	2.38	64-19-7	3.43	-	-	0.41
3	Butanal, 3-methyl-	3.20	590-86-3	1.02	-	-	-
4	2-Pentanone	3.54	107-87-9	-	0.32	-	-
5	2-Butanone, 3-methyl-	3.73	563-80-4	0.67	-	-	-
6	Pentanal	3.75	110-62-3	-	1.63	-	0.83
7	2-Pentenal, (E)-	4.97	1576-87-0	-	-	-	0.51
8	1-Pentanol	5.27	71-41-0	-	1.20	4.1	0.52
9	Hexanal	6.10	66-25-1	10.67	11.22	13.73	15.3
11	Pyrazine, methyl-	6.12	109-08-0	-	-	1.92	0.21
12	2-Pentanone, 4-hydroxy-	7.06	4161-60-8	4.02	-	-	-
13	2-Hexenal	7.67	505-57-7	-	0.18	-	0.66
14	2-Hexenal, (E)-	7.69	6728-26-3	-	0.17	-	1.20
15	1-Hexanol	8.19	111-27-3	-	7.44	34.76	4.28
16	2-Heptanone	8.27	110-43-0	-	-	9.15	3.91
17	Hydroperoxide, hexyl	8.87	4312-76-9	1.13	-	-	-
18	Heptanal	9.21	111-71-7	1.49	1.32	3.01	1.36
19	Pyrazine, 2,5-dimethyl-	9.49	123-32-0	-	-	6.82	1.84
20	2-Heptenal, (Z)-	10.15	57266-86-1	1.15	8.14	1.72	20.41
21	3-Pentenoic acid, 4-methyl-	10.93	504-85-8	49.72	8.1	-	-
22	1-Heptanol	11.48	111-70-6	-	-	-	1.66
23	1-Hepten-3-one	11.71	2918-13-0	-	-	-	0.04
24	1-Hepten-3-ol	11.78	4938-52-7	-	-	-	2.08
25	2-Octanone	12.09	111-13-7	-	7.90	-	-
26	Furan, 2-pentyl-	12.16	3777-69-3	-	-	4.33	12.89
27	2,4-Heptadienal, (E,E)-	12.25	4313-03-5	-	0.90	-	-
28	Octanal	12.50	124-13-0	-	-	9.67	5.82
29	Hexanoic acid	12.57	142-62-1	-	4.27	-	-
30	2,4-Heptadienal, (E,E)-	12.69	4313-03-5	-	2.23	-	-
31	5-Hepten-2-one, 6-methyl-	13.21	110-93-0	0.83	-	-	-
32	2-Nonenal, (Z)-	13.39	60784-31-8	1.18	-	-	-
33	1-Octanol	13.54	111-87-5	-	-	2.71	-
34	3-Octen-2-one, (E)-	13.61	18402-82-9	-	0.69	-	-
35	Decane	13.69	124-18-5	4.96	-	-	-
36	Pyrazine, 2-ethyl-3,5-dimethyl-	13.73	13925-07-0	-	-	2.00	-
37	2-Octenal, (E)-	14.21	2548-87-0	-	7.50	-	5.66
38	Nonane, 2,5-dimethyl-	14.49	17302-27-1	0.31	-	-	-
39	Decane, 2,6,7-trimethyl-	14.61	62108-25-2	0.81	-	-	-
40	Limonene	14.80	138-86-3	5.98	-	-	-
41	Heptanoic acid	14.99	111-14-8	-	1.80	-	-
42	o-Cymene	15.08	527-84-4	-	0.42	-	-
43	3-Octen-2-ol	15.33	76649-14-4	-	3.31	-	-
44	3,5-Octadien-2-one, (E,E)-	15.34	30086-02-3	-	-	-	2.72
45	Decane, 4-methyl-	16.05	2847-72-5	0.77	-	-	-
46	Decane, 2-methyl-	16.19	6975-98-0	0.88	-	-	-
47	Decane, 3-methyl-	16.43	13151-34-3	1.40	-	-	-
48	Nonanal	17.66	124-19-6	6.37	16.41	6.09	2.34
49	1-Nonanol	17.69	143-08-8	-	0.71	-	-
50	Octanoic acid	17.84	124-07-2	-	1.72	-	-
51	Dodecane	18.69	112-40-3	2.03	0.42	-	-
52	Decanal	18.73	112-31-2	1.20	0.95	-	0.49
53	2-Decenal, (E)-	20.29	3913-81-3	-	3.49	-	1.46
54	Nonanoic acid	20.58	112-05-0	-	2.54	-	-
55	2,4-Decadienal, (E,Z)-	21.81	25152-83-4	-	5.01	-	2.34

“-” means not detected.

**Table 6:** Changes in the color values of L*, a* and b* of the color sensor during storage at 60 °C for 50 days.

		10 d	20 d	30 d	40 d	50 d
CPO	L*	51.20 ± 1.68 ^c^	50.65 ± 0.19 ^c^	62.38 ± 1.18 ^a^	53.91 ± 0.76 ^b^	52.28 ± 1.71 ^bc^
	a*	21.76 ± 1.33 ^a^	22.70 ± 1.72 ^a^	22.54 ± 2.01 ^a^	10.40 ±0.83 ^b^	10.79 ± 0.53 ^b^
	b*	−1.71 ± 0.34 ^a^	−2.20 ± 0.61 ^a^	−2.77 ± 1.14 ^a^	−9.89 ± 0.64 ^c^	−8.10 ± 0.20 ^b^
	PV	0.15 ± 0.01 ^d^	0.21 ± 0.00 ^c^	0.20 ± 0.01 ^c^	0.26 ± 0.01 ^b^	0.31 ± 0.01 ^a^
Sensor Image		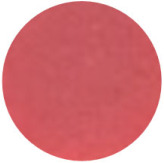	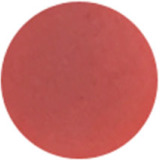	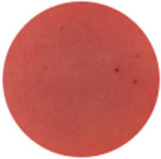	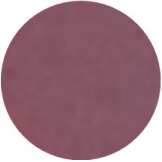	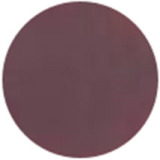
HPO	L*	44.43 ± 1.81 ^c^	46.90 ± 0.74 ^c^	63.92 ± 0.84 ^a^	53.25 ± 1.01 ^b^	54.98 ± 3.45 ^b^
	a*	18.48 ± 1.04 ^b^	20.24 ± 0.96 ^b^	25.84 ± 0.88 ^a^	14.76 ± 1.96 ^c^	14.59 ±2.88 ^c^
	b*	−1.61 ±0.15 ^a^	−1.51 ± 0.43 ^a^	−0.85 ±0.60 ^a^	−7.49 ± 1.00 ^b^	−6.63 ± 1.41 ^b^
	PV	0.09 ± 0.00 ^e^	0.14 ± 0.01 ^d^	0.18 ± 0.01 ^c^	0.25 ± 0.00 ^b^	0.30 ± 0.01 ^a^
Sensor Image		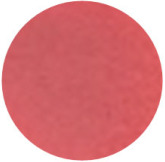	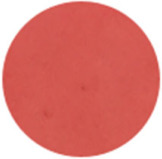	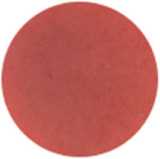	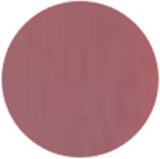	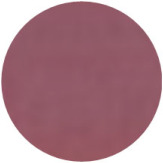

“a–c” means different lowercase letters indicate significant differences.

## Data Availability

The data presented in this study are available on request from the corresponding author.
